# AXINEO: AXIllary response to NEOadjuvant chemotherapy for breast cancer: can we predict response based on a biomarker panel?

**DOI:** 10.1007/s00404-025-08209-x

**Published:** 2025-11-18

**Authors:** Franziska Fick, Florian Lenz, Verena-Wilbeth Sailer, Achim Rody, Nikolas Tauber, Kerstin Muras, Natalia Krawczyk, Julika Ribbat-Idel, Franziska Hemptenmacher, Maggie Banys-Paluchowski

**Affiliations:** 1 Department of Gynecology and Obstetrics, Universitätsklinikum Schleswig-Holstein, Campus Lübeck, Ratzeburger Allee 160, 23562 Lübeck, Germany; 2 Department of Pathology, Universitätsklinikum Schleswig-Holstein, Campus Lübeck, Lübeck, Germany; 3 Department of Gynecology and Obstetrics, Universitätsklinikum Düsseldorf, Düsseldorf, Germany

**Keywords:** Nodal positive breast cancer, Neoadjuvant chemotherapy, Prognostic biomarkers, Pathological complete response

## Abstract

**Background:**

Up to 60% of breast cancer patients achieve pathological complete response (pCR) and factors associated with breast pCR have been extensively investigated. In patients with initially node-positive disease predicting axillary response to treatment remains challenging. Our study examines a biomarker panel assessed on core-biopsy lymph-node metastatic tissue with the goal to establish predictive markers for nodal positive breast cancer.

**Materials and methods:**

Forty women with core biopsy-proven node-positive breast cancer scheduled to receive neoadjuvant treatment at the certified Breast Cancer Center of the University Hospital Schleswig–Holstein Campus Lübeck were included. The expressions of CAIX, PD-L1, TROP2, MSH2, MSH6, MLH1, and PMS2 as well as p53 mutation were assessed. Biomarkers were chosen based on their association with tumorigenesis and tumor progression. Statistical analysis was performed using SPSS 29. This investigator-initiated study was supported by a research grant from Gilead (Gilead Förderprogramm).

**Results:**

Higher CAIX levels were associated with triple-negative and Her2-positive receptor status (*p* = 0.003), Ki67 ≥ 50% in breast core biopsy (*p* = 0.005), as well as postmenopausal status (*p* = 0.007). P53 mutation was more frequent in G3 tumors (*p* = 0.025). All lymph-node metastases were microsatellite stable (MSS). None of the markers could significantly predict pathological response (complete, breast, or nodal).

**Conclusion:**

Our study shows upregulated CAIX in lymph-node metastasis frequently occurs in aggressive and highly proliferative tumors. However, none of the examined biomarkers could predict nodal response to therapy. Further research is necessary to better identify patients most likely to achieve nodal response through neoadjuvant chemotherapy.

**Supplementary Information:**

The online version contains supplementary material available at 10.1007/s00404-025-08209-x.

## What does this study add to the clinical work

**Table:** 

The study examines the impact of the molecular profile of metastatic lymph node tissue on the response to neoadjuvant therapy for node-positive breast cancer.	
CAIX levels were associated with receptor status and Ki67 and P53 mutations were more frequent in G3 tumors.	
Further research of CAIX expression might help to better identify patients most likely to achieve nodal response after neoadjuvant treatment and be therefore suitable candidates for surgical de-escalation.	

## Introduction

Breast cancer (BC) is the most common malignancy in women worldwide [1]. In Germany, one in five patients receive neoadjuvant chemotherapy (NACT) before definitive surgery [2]. Depending on the subtype and extent of disease, NACT is able to eradicate all invasive tumor cells (pathological complete response = pCR) in up to 60% of patients with early BC and factors associated with breast pCR have been extensively investigated. Regarding nodal response, recent data show that approximately 50–60% of patients initially cN + have no vital tumor cells in axillary lymph nodes following NACT. Importantly, axillary response to therapy guides treatment choices regarding surgical therapy of the axilla [3–8]. Patients with cN + → ycN0 conversion are recommended de-escalated procedures, such as targeted axillary dissection or sentinel node biopsy, while whose with persistently suspicious lymph nodes are usually offered axillary lymph-node dissection, associated with increased risk of arm morbidity and lymph edema [9–13]. However, predicting axillary response to treatment remains challenging, and imaging such as axillary ultrasound often fails to accurately detect axillary residual disease [14]. Therefore, tools allowing for a reliable identification of patients most likely to reach axillary pCR are urgently needed.

Beyond established predictive factors such as tumor size, number of positive lymph nodes, hormone receptor and Her2 status, grading and ki67, assessing tissue-based biomarkers may further improve response prediction.

The present study aimed at examining a panel of biomarkers assessed on core biopsy of lymph-node metastatic tissue with the goal to establish predictive markers for treatment response to neoadjuvant chemotherapy in node-positive breast cancer. The following biomarkers were chosen based on their association with tumorigenesis and tumor progression: p53 expression status, TROP 2 (tumor-associated calcium signal transducer 2), PD-L1 (Programmed Cell Death 1 Ligand 1), CAIX (Hypoxia-regulated carbonic anhydrase IX), and MSI (microsatellite instability).

## Methods

We analyzed core biopsy specimens of lymph-node metastases of 40 female patients treated at the certified Breast Center at the University Hospital Schleswig–Holstein between 2018 and 2024. Patients were chosen retrospectively according to their nodal pCR (50% nodal pCR, 50% nodal non-pCR). We defined complete pCR as ypT0 and ypN0. Patients were selected retrospectively due to their postoperative pCR result in a consecutive manner. The study was supported by a research grant from Gilead (*Gilead* Förderprogramm). The study was approved by the local ethical committee and only patients who consented to the use of their data and archived tissue were included. Inclusion criteria are shown in Table 1.

**Table 1:** Inclusion criteria of the AXINEO study

Inclusion criteria
Biopsy proven node-positive breast cancer scheduled to receive neoadjuvant treatment with chemotherapy
Sufficient lymph-node tissue (core-biopsy) for pathological analysis
Broad consent signed by the patient [broad consent = standardized informed consent for the secondary use of identifiable private information and biospecimens for future, unspecified research purposes]

Immunohistochemical evaluation of core biopsy specimens

The expression of p53, CAIX, PD-L1, TROP2, MSH2, MSH6, MLH1, and PMS2 was assessed using immunohistochemistry on 4 µm thin sections. A positive control was provided for each marker (Table 2).

**Table 2:** Targets and used antibodies

Antibody target	Name	Dilution	Manufacturer	Positive control
MLH1	MLH1	RTU*	Ventana (Roche)	Colon
MSH2	MSH2	RTU	Ventana (Roche)	Colon
MSH6	MSH6	RTU	Ventana (Roche)	Colon
PMS2	PMS2	RTU	Ventana (Roche)	Colon
p53	p53	RTU	Ventana (Roche)	Tonsil
CA IX	Carbonic anhydrase IX	RTU	Ventana (Roche)	Gastric mucosa
TROP2	TROP2	1:500	abcam	Breast cancer
PD-L1	PD-L1	1:50	Dako	Tonsil

*RTU ready to use

The p53 staining result was interpreted as previously described in ovarian carcinomas [15] and was indicated as either mutational-type or wild-type. PD-L1 status was evaluated as the proportion of tumor cells with any membrane-bound staining reaction and was reported as TPS. Due to the lack of differentiation between tumor-associated immune cells and local immune cells of the lymph-node tissue, the CPS was not further evaluated. The mismatch-repair proteins were considered intact/preserved if all tumor cells showed a consistent, strong nuclear staining. In contrast, a complete lack of nuclear staining was declared as a loss, indicating a mismatch-repair deficiency. The stained slides of CAIX and TROP2 were scanned using Ventana DP200 (Roche) at 40 × magnification. QuPath software (version 0.4.4) was used to visualize the staining for CAIX and TROP2. A pathologist manually marked the tumor cells and set the parameters for cell detection as well as thresholds for classification in negative (0), weakly (1+), moderately (2+), and strongly (3+) positive tumor cells. Subsequently the immunohistochemical expression was quantified by the software providing the H-score. The mean value of the H-Scores of each tumor sample was used for statistical analysis.

### Statistical analysis

We used SPSS Version 29 for statistical analyses. First, the data were examined for the presence of a normal distribution. Depending on the result, the H-scores of the respective cohorts were compared using the Mann–Whitney and Kruskal–Wallis tests (CAIX and PDL1), Chi-squared test (P53), or the ANOVA (TROP2). The p53 status was reported for each tumor sample as mutation type or wild type. In analogy, the tumor samples were classified as showing an intact or lost staining for the mismatch-repair proteins. The frequencies of the respective type in the two cohorts resulted in nominal scaled data and were statistically analyzed using the Chi-squared test. The significance level was set at *p* < 0.05. Primary endpoint was the correlation with therapy response, defined as pCR (complete, breast, and nodal).

## Results

### Tumor biology and patients characteristics

Median age at diagnosis was 53 years (SD 12.3, range: 28–77 years). 17 out of 40 (42.5%) patients were premenopausal, one (2.5%) perimenopausal, and 22 (55%) postmenopausal at diagnosis. The median BMI was 26.1 (range 19.2–44.1). All tumors had at least a G2 grading, 75% were G3. Median tumor size was 27.7 mm (SD 17.3). One patient had an inflammatory breast cancer. The median number of suspicious lymph nodes at time of diagnosis was two (SD 1.2, range 1–5).

**Fig. 1:**
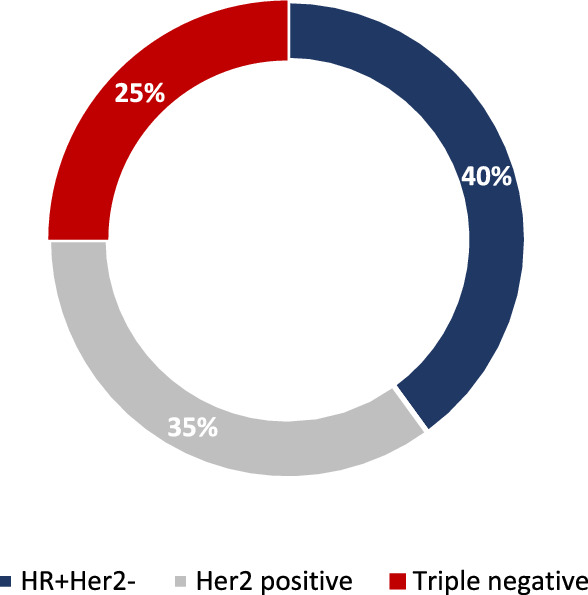
Receptor status of included patients

The most common subtype, assessed on primary tumor, was hormone receptor (HR) positive Her2 negative (16; 40%), followed by Her2 positive (14; 35%) and triple-negative breast cancer (TNBC) (10 patients, 25%, Fig. 1). Among patients with Her2 positive disease, 8 had HR-positive and 6 HR negative tumors (Supplementary Table 2). One patient presented with a bifocal cancer, with one lesion HR-positive Her2 negative and the other one Her2 positive. Median Ki67 was 50% (range 15–99%).

24 out of 40 (60%) patients underwent germline genetic testing at the certified Center for Familial Breast and Ovarian Cancer at the UKSH Campus Lübeck. Out of these, six (25% of all tested patients and 15% of the total cohort) were carriers of (likely) pathogenic variants (1 × BRCA1, 3 × BRCA2, 1 × CHEK2, 1 × PTEN).

The comparison of the study cohort characteristics and the population treated at the certified Breast Cancer Center of UKSH Campus Lübeck is shown in Supplementary Table 1. Approx. 600 patients with newly diagnosed breast cancer are treated at our institution each year. During the study period (2018–2024), 290 patients received NACT, including 136 with node-positive disease at time of diagnosis. The proportion of different subtypes of patients included in the present analysis and all patients treated within study period was similar. Not all of the 136 patients with node-positive breast cancer gave their broad consent to future research with data and tissue. Further, the grant obtained from the *Gilead Förderprogramm* limited the number of assays performed to 40 patients. These were selected retrospectively according to the inclusion criteria shown in Table 1.

All 40 patients underwent neoadjuvant therapy for node-positive breast cancer. The systemic therapy regimen was recommended in the multidisciplinary tumor board according to the national and institutional guidelines at time of presentation. 26 out of 40 (65.0%) patients completed NACT according to schedule without dose reductions. The remaining 14 (35.0%) patients experienced minor or major adverse events (e.g., peripheral neuropathy, diarrhea, hematological toxicity, or acute kidney injury) and either underwent dose reduction or discontinued therapy or. Details of chemotherapy regimens administered can be found in Supplementary Table 2.

Specifically, nine (22.5%) patients discontinued treatment after receiving several cycles of therapy. Two patients with TNBC discontinued pembrolizumab after three administrations due to severe autoimmune thyroiditis (grade 3) but received full chemotherapy treatment. In one patient with TNBC, NACT was terminated after 12 × nab-paclitaxel 125 mg/m^2^ + carboplatin AUC 1.5, followed by 3 × epirubicin/cyclophosphamide due to clinical progression; this is missing one cycle of EC. Three patients with Her2-positive disease discontinued treatment: one received three cycles of TCbHP regimen and discontinued due to acute kidney injury, another one stopped after receiving five out of six cycles due to diarrhea and hypokalemia, and the third one discontinued pertuzumab due to severe diarrhea. One patient was scheduled to receive dose-dense ETC regimen and refused the last administration of cyclophosphamide. Two patients with HR-positive HER2-negative disease received four cycles of EC but discontinued paclitaxel, due to peripheral neuropathy (*n* = 1) and pneumonitis (*n* = 1).

In five patients (12.5%), one or more dose reductions were necessary due to treatment-related toxicity. Specifically, 80% dose reduction of cyclophosphamide during the dose-dense ETC regimen was required in one patient, 80% dose reduction of paclitaxel due to peripheral neuropathy in three patients, and one patient received 80% carboplatin in the TCbHP protocol due to diarrhea.

### Surgical treatment

Twenty-one patients (52.5%) received a breast-conserving therapy and 19 a mastectomy (47.5%). The most common axillary procedure was axillary lymph-node dissection performed in 20 patients (50%), followed by a targeted axillary dissection (TAD) in 17 patients (42.5%) and sentinel lymph-node biopsy in three patients (7.5%). Out of 17 patients who received a TAD, four (23.5%) underwent completion axillary lymph-node dissection. In summary, 24 patients received axillary dissection in total (20 in an upfront setting and 4 as a secondary procedure following TAD).

### Response to therapy

**Fig. 2:**
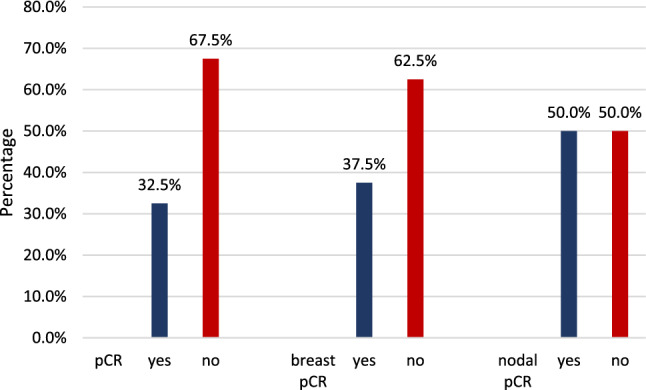
PCR rates in the study cohort (complete, breast, and nodal)

Patients were selected for this study based on their nodal response to treatment (ycN0 vs. ycN+). 13 out of 40 patients (32.5%) had a complete pCR, defined as ypT0 ypN0. One patient (2.5%) had a non-invasive tumor rest (ypTis ypN0). The remaining 26 patients (65.0%) had invasive tumor rest in the breast and/or lymph nodes (Fig. 2).

**Fig. 3:**
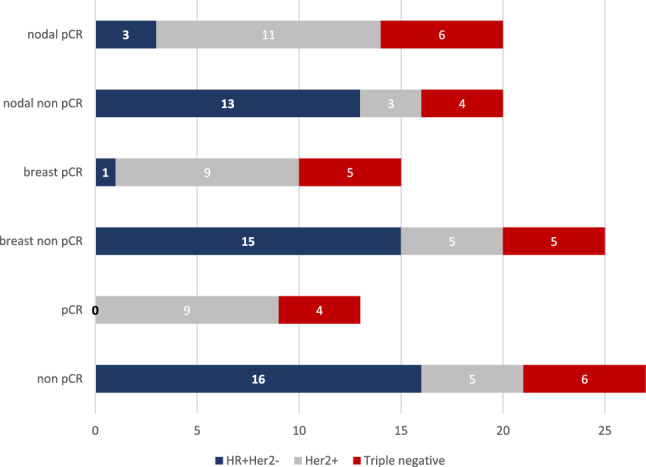
PCR rates according to the receptor status of the primary tumor

The pCR rates (complete, breast, and nodal) differed between tumors with different receptor status (complete pCR: *p* < 0.001, breast pCR: *p* = 0.003, nodal pCR: *p* = 0.004, Fig. 3). The lowest pCR rate was observed in HR-positive Her2 negative breast cancers (complete pCR: 0%, breast pCR: 6.25%, nodal pCR: 18.75%), compared to Her2 positive (complete pCR: 64.29%, breast pCR: 64.29%, nodal pCR: 78.57%) and triple-negative tumors (complete pCR: 40%, breast pCR: 50%, nodal pCR: 60%).

Based on the recent data, BMI was also included in the analysis [16]. Median BMI in patients who reached nodal pCR was numerically higher (25.7, range 21.1–40.2), compared to 23.9 (range 19.2–44.1) in patients with non-pCR. Median BMI in patients with TNBC was 23.1 (range 19.2–27.5). Among these, 75% of patients with normal BMI (< 25) were in the nodal pCR cohort and 25% in the non-pCR group.

### Biomarker analysis

In our cohort, none of the markers tested on the lymph-node core-biopsy specimen correlated significantly with the response to chemotherapy (Table 3). Further, no significant differences in the tested markers were shown between germline mutation carriers and non-carriers.

**Table 3:** Results of the biomarker panel assessed on the core-biopsy lymph-node metastatic tissue and clinical–pathological factors

	Total (*n*)	TROP 2	TROP2 high*	P53 mutation	PD-L1	PD-L1 ≥ 1%	CAIX	CAIX high*
Total number of analyzed patients (*n*)	40	39	20 Median: 171.0	40	38	9	39	20 Median: 0.025
pCR^a^								
Yes	13	12	6 (50%)	9 (69.2%)		1 (8.3%)		8 (66.7%)
No	27	27	14 (51.9%)	15 (55.6%)		8 (30.8%)		12 (44.4%)
*p* value		0.571	0.915	0.408	0.756	0.130	0.070	0.200
Breast pCR^b^								
Yes	15	14	8 (57.1%)	10 (66.7%)		2 (14.3%)		9 (64.3%)
No	25	25	12 (48%)	14 (56%)		7 (29.2%)		11 (44%)
*p* value		0.315	0.584	0.505	0.790	0.298	0.251	0.224
Nodal pCR								
Yes	20	19	9 (47.4%)	14 (70%)		4 (22.2%)		12 (63.2%)
No	20	20	11 (55%)	10 (50%)		5 (25%)		8 (40%)
*p* value		0.562	0.634	0.197	0.752	0.841	0.166	0.148
Age								
≥ 53 years	20	19	9 (47.4%)	13 (65%)		7 (36.8%)		14 (73.7%)
< 53 years	20	20	11 (55%)	11 (55%)		2 (10.5%)		6 (30%)
*p* value		0.506	0.421	0.519	0.057	0.056	**0.033**	**0.006**
Grading^c^								
G2	10	10	3 (30.0%)	3 (30%)		1 (11.1%)		3 (30%)
G3	30	29	17 (56.7%)	21 (70%)		8 (27.6%)		17 (58.6%)
*p* value		0.078	0.118	**0.025**	0.336	0.310	0.272	0.118
Ki67^3^								
≥ 50%	20	20	10 (50%)	15 (75%)		6 (33.3%)		12 (60%)
< 50%	20	19	10 (52.6%)	9 (45%)		3 (15%)		8 (42.1%)
*p* value		0.288	0.869	0.053	0.775	0.184	0.235	0.264
Subtype								
NST	36	35	17 (48.6%)	20 (57.1%)		9 (26.5%)	37	15 (42.9%)
Other	4	4	1 (50%)	3 (75%)		0	2	3 (75%)
*p* value		0.905	0.998	0.787	–	0.331	**0.021**	0.197
Menopausal status								
Pre/perimenopausal	18	18	9 (50%)	10 (55.6%)		2 (11.1%)		5 (27.8%)
Postmenopausal	22	21	11 (52.4%)	14 (45.5%)		7 (33.3%)		15 (71.4%)
*p* value		0.903	0.882	0.604	0.130	0.120	**0.028**	**0.007**
Receptor status								
HR + HER2-	16	16	7 (43.8%)	6 (37.5%)		2 (13.3%)		3 (18.8%)
HER2 positive	14	13	6 (46.2%)	10 (71.4%)		3 (21.4%)		9 (69.2%)
Triple-negative	10	10	7 (70%)	8 (80%)		4 (44.4%)		8 (80%)
*p* value		0.415	0.386	0.055	0.247	0.215	0.263	**0.003**
Germline mutation^d^								
Yes	6	6	3 (50%)	3 (50%)		1 (20%)		3 (50%)
No	16	15	7 (46.7%)	4 (25%)		4 (25%)		6 (40%)
No data/not tested	18	18	10 (55.6%)	9 (50%)		4 (23.5%)		11 (61.1%)
*p* value		0.592	0.877	0.287	0.925	0.974	0.305	0.481

*p* values < 0.05 are shown in bold; since all specimens were microsatellite stable, MSI status was not included in the table

*Defined as expression level above median

^a^Defined as ypT0 ypN0

^b^Defined as ypT0

^c^Breast biopsy

^d^Germline mutation tested with the German Consortium for Familial Breast and Ovarian Cancer Panel

#### TROP2

TROP2 expression (H-Score) was analyzed in 39 patients with an average level of 177.85, median 171.0, with a standard deviation (SD) of 59.21; range 1.4–290.0. There were no significant differences shown for the TROP2 H-Score and the different parameters tested (Table 3). Since there are no established cut-off levels for TROP2 assessment on lymph-node metastatic tissue, TROP2-high was defined as TROP2 expression level above the median (171.0). There were no significant associations with other factors nor response to treatment. There was no correlation between high CAIX expression (defined as levels above median) and high TROP2 score (*p* = 0.634).

#### P53

The p53 protein expression was described as binary value (mutated/wild type). The tumors of 24 patients (60%) showed a p53 mutational-type-expression and 16 (40%) showed p53 wild-type expression. P53 mutation was significantly more frequent in G3 tumors (*p* = 0.025). 14 out of 20 patients (70.0%) with nodal pCR had a p53 mutation in the lymph-node metastatic tissue, compared to 10 out of 20 (50.0%) with nodal non-pCR. There were no other significant correlations found. With regard to receptor status, HR-positive Her2-negative tumors showed p53 mutations in 37.5% (*n* = 6) of cases, whereas Her2-positive and triple-negative tumors harbored p53 mutations in 71.4% (*n* = 10) and 80% (*n* = 8) of cases, respectively (*p* = 0.055).

#### PD-L1

PD-L1 status could be successfully analyzed in 38 patients. The average value was 1.46% with a range from 0 to 10% and a standard deviation of 1.92%. No differences were found for PD-L1 between subgroups. Older patients were numerically more likely to have PD-L1 ≥ 1% in lymph-node metastasis than younger patients (*p* = 0.057).

#### CAIX

CAIX expression levels were 6.106 in average, median 0.025, with a standard deviation (SD) of 15.96; range 0.0 to 69.15. Higher CAIX levels assessed in the lymph-node core-biopsy were associated with triple-negative and Her2-positive receptor status (*p* = 0.003), Ki67 ≥ 50% (assessed in breast core biopsy, *p* = 0.005), as well as postmenopausal status (*p* = 0.007). 63.2% of patients who achieved nodal pCR had above-median CAIX expression (CAIX high) levels, compared to 40% among patients with non-pCR (*p* = 0.148). 80% (*n* = 8) of the triple-negative breast cancers had CAIX expression levels above median (*p* = 0.003), compared to 69.2% among the Her2 + subtype (*n* = 9) and 18.8% (*n* = 3) of the HR + Her2- group. We found significantly higher CAIX expressions in older and postmenopausal patients. The median for postmenopausal women was 0.670 and 0.035, respectively, for pre/perimenopausal patients (*p* = 0.028). Matching this result CAIX expression levels were also higher in patients ≥ 53 years old (average 6.303 vs. 5.919; SD 16.29 vs. 16.06; *p* = 0.033). In the CAIX high group, 14 patients (73.7%) were ≥ 53 years old, compared to 6 patients (30%) in the CAIX low group (*p* = 0.006).

#### MSI

All tumors were microsatellite stable (testing was performed with MSH2, MSH6, MLH1, and PMS2 immunohistochemistry), so no further analysis could be made.

## Discussion

To the best of our knowledge, this is the first comprehensive biomarker analysis of lymph-node metastatic tissue in patients receiving neoadjuvant chemotherapy. The biomarkers were chosen based on their association with tumorigenesis and tumor progression. Consecutive patients were selected based on their response to therapy in the lymph nodes. As expected, in the present analysis, more patients in the nodal pCR group had triple-negative and HER2-positive tumors, compared to patients with nodal non-pCR. This goes along with research findings of the meta-analysis from 2020 which showed pCR rates to be lower in HR-positive breast cancers [17].

Hypoxia-regulated carbonic anhydrase IX (CAIX) is a hypoxia-inducible molecular marker. The majority of studies suggest that CAIX can serve as a biomarker and therapeutic target in different tumor types [18, 19]. CAIX can promote tumorigenesis and is associated with a more aggressive phenotype of cancer cells [20]. Ong et al. analyzed specimens of more than 300 TNBC patients and found out that increased CAIX protein levels are independently associated with poor survival [21, 22]. Interestingly, in our study, 80% of patients with triple-negative breast cancers had a CAIX expression in lymph-node metastasis above median (CAIX high), compared to 69.2% of the Her2 + subtype and 18.8% of the HR + Her2-negative group (*p* = 0.003). This is in accordance with previous studies, which reported a significantly higher expression of CAIX in triple-negative breast cancer [21]. Furthermore, overexpression of CAIX protein in TNBC is associated with a BRCA1 mutant signature and loss of BRCA1 function [22]. As there was only one patient with BRCA1 mutation in our study, further analyses were not possible. The correlation between CAIX and BRCA1 mutation, clearly associated with triple-negative subtype, should be investigated in future studies.

One critical aspect that is worth noting in this context is the relatively high inter-observer variability of CAIX expression and its dependence on the quality of biopsies [23]. Techniques to standardize histopathological assessment and lower inter-observer variability still need to be optimized to make the marker more comparable. Currently, research on CAIX fluoroscopy in single-photon emission computerized tomography (SPECT), positron emission tomography (PET), and near-infrared fluorescence imaging (NIRF) is ongoing. Hypothetically, these imaging modalities could be used in nodal positive breast cancer patients to determine CAIX expression in the future [24].

Regarding potential therapeutic consequences, CAIX-based agents like the CAIX-specific monoclonal antibody girentuximab have been evaluated in a phase III clinical trials in high-risk renal cell carcinoma [25]. Currently, no studies in breast cancer patients are available. Implementation of CAIX-based treatment could potentially enable new therapeutic strategies for high-risk breast cancer patients (e.g., triple negative, Ki67 ≥ 50%).

In the present study, we show higher TROP2 expression in lymph-node metastasis in patients with TNBC. TROP2 is a membrane protein involved in tumor progression by actively interacting with several key signaling pathways associated with cancer development and plays an important role in tumor growth, invasion, treatment resistance, and metastasis. TROP2-targeted drugs are available such as sacituzumab govitecan [26]. Further, high TROP2 expression correlates with poorer response and drug resistance [27, 28]. Previous studies have shown TROP2 expression to be heterogeneous among breast cancer subtypes [29, 30], with higher expression in triple-negative breast cancer. However, in contrast to our study, these analyses were performed on breast specimens [29, 30].

In our study, more patients with nodal pCR had p53 mutations in their lymph nodes (70 vs. 50%, *p* = 0.197) with further investigation in larger cohorts needed. P53 mutations were also more common in case of aggressive tumors (such as G3). We could also observe a potential difference in receptor status for p53 mutations. Accordingly, HR + Her2-negative cases showed p53 mutations in the lymph node in 37.5%, whereas Her2-positive and triple-negative patients showed p53 mutations in 71.4% and 80%, respectively (*p* = 0.055). P53, as a transcription factor, plays an important regulatory role in the multitude of cellular processes. Genotoxic stress leads to activation of p53 to facilitate DNA repair, cell-cycle arrest, and apoptosis. P53 has been shown to be an important biomarker in endometrial cancer but is not one of the standard markers in breast cancer [31], although it is the most frequently mutated gene in breast cancers (up to 30%, subtype depending). The evidence on the prognostic value of p53 is not conclusive and seems to depend on hormonal status and prior treatment [32].

MSI is an established marker for various tumors, such as colorectal and endometrial cancer, and is frequently used as a predictive factor for immune checkpoint inhibitor therapy [33]. In breast cancer, however, the prevalence of microsatellite instability is low (1.8% or less) [34–36]. This is in line with our study. All lymph-node metastatic specimens analyzed were microsatellite stable. Nevertheless, MSH2, MSH6, MLH1, and PMS2 are part of extended germline testing in BC patients using the TruRisk©-Panel at German Centers for Familial Breast and Ovarian Cancer which currently includes ATM, BRCA1, BRCA2, BRIP1, CDH1, CHEK2, EPCAM, MLH1, MSH2, MSH6, PALB2, PMS2, PTEN, RAD51C, RAD51D, STK11, and TP53 [37].

One limitation of our study is the moderate number of patients (*n* = 40). Some of the initially planned assays such as PIK3CA somatic mutational analysis were not possible because of the limited amount of core-biopsy tissue specimen available. As the tests were conducted on lymph-node metastatic core biopsies, we also cannot exclude intratumoral heterogeneity. This applies, for example, to PD-L1-status, for which high variability in expression within the same tumor has already been demonstrated in breast cancer [38]. Because of the retrospective selection of patients according to their nodal response, there might be a selection bias.

Further studies should focus on CAIX expression and TP53 mutational status as well as possibly further potential biomarkers to improve the selection of patients most likely to achieve axillary response.

## Summary

Among the biomarkers analyzed, numerical differences in the expression levels of CAIX and tissue mutational status of P53 were observed between patients with nodal pCR and non-pCR. Our study shows that upregulation of hypoxemia marker CAIX in the lymph-node metastatic tissue before start of systemic therapy is significantly associated with a more aggressive tumor subtype. Further research is necessary to better identify patients most likely to achieve nodal response after neoadjuvant treatment, and thus best eligible for surgical de-escalation, e.g., targeted axillary dissection or sentinel node biopsy alone instead of full axillary lymph-node dissection.

## Supplementary Information

Below is the link to the electronic supplementary material.

Supplementary file1 (DOCX 20 KB)

## Data Availability

No datasets were generated or analyzed during the current study.
