# Supplementary material for: AXINEO: AXIllary response to NEOadjuvant chemotherapy for breast cancer: can we predict response based on a biomarker panel?
Source: Arch Gynecol Obstet. 2025 Nov 18;312(6):2211–9. doi: 10.1007/s00404-025-08209-x (PMC12705802; doi:10.1007/s00404-025-08209-x)
Supplement: Supplementary file 1 — Supplementary file1 (DOCX 20 KB) [file 404_2025_8209_MOESM1_ESM.docx]

**Supplementary Table 1.** Comparison of patient characteristics between patients included in the AXINEO study and patient population treated at the Breast Cancer Center UKSH Campus Lübeck between 2018 and 2024.

|  | **Study cohort** | **Patients with node-positive breast cancer treated with NACT during study period** |
| --- | --- | --- |
| Number of patients | 40 | 136 |
| Mean age | 53; standard deviation 12.5 | 53, standard deviation 13.0 |
| Receptor status  HR pos HER2 neg  HER2 pos  TNBC | 40%  35%  25% | 34%  34%  32% |

## Neoadjuvant Chemotherapy (NACT)

**Supplementary Table 2.** Details of tumor stage, response to treatment, regimen administered and course of neoadjuvant therapy.

| **Patient number** | **Age** | **Clinical tumor stage** | **Receptor status** | **Neoadjuvant chemotherapy** | **NACT administered as planned** | **Breast pCR** | **Nodal pCR** |
| --- | --- | --- | --- | --- | --- | --- | --- |
| 1 | 52 | cT2 cN1 G3 | ER 90%  PR 70% Her2 0 Ki67 5% | 4 x epirubicin 90 mg/m^2^ + cyclophosphamid 600 mg/m^2^ dd q2w, followed by 12 x paclitaxel 80 mg/m^2^ weekly | Yes | No | No |
| 2 | 34 | cT2 cN1 G2 | ER 100% PR 70% Her2 3+ Ki67 30% | 6 x TCbHP (docetaxel, carboplatin, trastuzumab, pertuzumab) | Yes | No | No |
| 3 | 67 | cT2 cN2 G3 | ER 0%  PR 0% Her2 low (2+, ISH neg.)  Ki67 85% | 12 x nab-paclitaxel 125 mg/m^2^ + carboplatin AUC 1.5 q1w, followed by 3 x epirubicin 90 mg/m^2^ + cyclophosphamid 600 mg/m^2^ dd q2w + pembrolizumab q3w | No:  stop due to progression after C3 EC | No | Yes |
| 4 | 46 | cT3 cN3 G3 | ER 5%  PR 0% Her2 low (1+) Ki67 99% | 12 x nab-paclitaxel 125 mg/m^2^ + carboplatin AUC 1.5 q1w, followed by 4 x epirubicin 90 mg/m^2^ + cyclophosphamid 600 mg/m^2^ dd q2w + 3 x pembrolizumab q3w | No:  discontinuation of immunotherapy after 3 cycles due to autoimmunthyreoditis, full dose of chemotherapy completed | No | No |
| 5 | 67 | cT1 cN1 G2 | ER 100% PR 30% Her2 low (2+, ISH neg) Ki67 20-30% | 4 x epirubicin 90 mg/m^2^ + cyclophosphamid 600 mg/m^2^ dd q2w, followed by 12x Paclitaxel 80mg/m2 q1w in combination with trastuzumab (loading dose 8 mg/kg, maintainance 6 mg/kg) + pertuzumab (loading dose 840mg, maintainance dose 420mg) | Yes | No | No |
| 6 | 55 | cT1 cN1 G3 | ER 70%  PR 50% Her2 pos (2+, ISH pos.) Ki67 30% | 6 x TCbHP (docetaxe 75 mg/m², carboplatin AUC 6, trastuzumab loading dose 8 mg/kg, maintainance 6 mg/kg , pertuzumab loading dose 840mg, maintainance dose 420mg) q3w | No:  discontinuation Pertuzumab due to acute kidney failure grade III linked to severe diarrhea grade III | No | No |
| 7 | 62 | cT1 cN2 G2 | ER 100% PR 100% Her2 low (1+) Ki67 10% | E-T-C  3x epirubicin (E) 150 mg/m2 q2w, followed by  3x paclitaxel (T) 225 mg/m2 q2w, followed by  3x cyclophosphamide (C) 2500 mg/m2 q2w | No:  dose reduction to 80% cyclophosphamide C1 due to anemia grade III | No | No |
| 8 | 56 | cT2 cN1 G2 | ER 100% PR 30% Her2 low (1+) Ki67 10% | 4x epirubicin 90mg/m2 + cyclophosphamide 600mg/m2 dd q2w, followed by 12x paclitaxel 80mg/m2 q1w | Yes | No | No |
| 9 | 50 | cT2 cN2 G2 | ER 100% PR 100% Her2 low (1+) Ki67 30-40% | E-T-C  3x epirubicin (E) 150 mg/m2 q2w  followed by  3x paclitaxel (T) 225 mg/m2 q2w  followed by  3x cyclophosphamide (C) 2500 mg/m2 q2w | No:  patient refused C3 cyclophosphamide due to fatigue | No | No |
| 10 | 41 | cT4d cN3a G3 | ER 100% PR 100% Her2 0 Ki67 30% | E-T-C  3x epirubicin (E) 150 mg/m2 q2w  followed by  3x paclitaxel (T) 225 mg/m2 q2w  followed by  3x cyclophosphamide (C) 2500 mg/m2 q2w | No:  80% taxol due to neuropathy grade II | No | No |
| 11 | 46 | cT2 cN3 G3 | ER 30%  PR 0% Her2 3+ Ki67 15% | 4 x epirubicin 90 mg/m^2^ + cyclophosphamid 600 mg/m^2^ dd q2w, followed by 12x nab-paclitaxel 80mg/m2 q1w in combination with trastuzumab (loading dose 8 mg/kg, maintainance 6 mg/kg) + pertuzumab (loading dose 840mg, maintainance dose 420mg) | No:  C8-C12 dose reduction taxol 80% due to peripheral neuropathy grade II | No | No |
| 12 | 70 | cT1 cN2a G2 | ER 60%  PR 30% Her2 0 Ki67 20% | 4x epirubicin 90mg/m2 + cyclophosphamid 600mg/m2 dd q2w, followed by 12x paclitaxel 80mg/m2 q1w | No:  discontinuation after C7 due to peripheral neuropathy grade III | No | No |
| 13 | 70 | cT4 cN2a G3 | ER 40%  PR <10% Her2 0 Ki67 50-60% | E-T-C  3x epirubicin (E) 150 mg/m2 q2w  followed by  3x paclitaxel (T) 225 mg/m2 q2w  followed by  3x cyclophosphamide (C) 2500 mg/m2 q2w | No:  C3 taxol 80% | No | No |
| 14 | 74 | cT2 cN2a G3 | ER 40%  PR <10% Her2 0 Ki67 50-60% | E-T-C  3x epirubicin (E) 150 mg/m2 q2w  followed by  3x paclitaxel (T) 225 mg/m2 q2w  followed by  3x cyclophosphamide (C) 2500 mg/m2 q2w | Yes | No | No |
| 15 | 39 | cT4d cN1a G3 | ER 100%  PR 50% Her2 low (2+ ISH neg.)  Ki67 25% | 12 x nab-paclitaxel 125 mg/m^2^ + carboplatin AUC 1.5 q1w, followed by 4 x epirubicin 90 mg/m^2^ + cyclophosphamid 600 mg/m^2^ dd q2w + 3 x pembrolizumab q3w | Yes | Yes | No |
| 16 | 47 | cT2 cN1 G3 | ER 80%  PR 60% Her2 0  Ki67 30% | 4x epirubicin 90mg/m2 + cyclophosphamid 600mg/m2 dd q2w, followed by 12x paclitaxel 80mg/m2 q1w | Yes | No | No |
| 17 | 54 | cT1 cN2 G3 | ER 90%  PR 30% Her2 low (2, ISH neg.)  Ki67 70% | 4x epirubicin 90mg/m2 + cyclophosphamid 600mg/m2 dd q2w, followed by 12x paclitaxel 80mg/m2 q1w | No:  discontinuation due to pneumonitis grade II after c6 pqclitaxel | No | No |
| 18 | 42 | cT1 cN1 G2 | ER 90%  PR 100% Her2 low (1+)  Ki67 20% | 4x epirubicin 90mg/m2 + cyclophosphamid 600mg/m2 dd q2w, followed by 12x paclitaxel 80mg/m2 q1w | Yes | No | No |
| 19 | 60 | cT2 cN3 G3 | ER 0%  PR 0% Her2 low (1+)  Ki67 70% | 12 x nab-paclitaxel 125 mg/m^2^ + carboplatin AUC 1.5 q1w, followed by 4 x epirubicin 90 mg/m^2^ + cyclophosphamid 600 mg/m^2^ dd q2w + 3 x pembrolizumab 200mg q3w | Yes | Yes | No |
| 20 | 36 | cT2 cN1 G3 | ER 0%  PR 0% Her2 low (2+, ISH neg.)  Ki67 60% | 12 x nab-paclitaxel 125 mg/m^2^ + carboplatin AUC 1.5 q1w, followed by 4 x epirubicin 90 mg/m^2^ + cyclophosphamid 600 mg/m^2^ dd q2w + 3 x pembrolizumab 200mg q3w | Yes | No | No |
| 21 | 73 | cT1 cN1 G3 | ER 0%  PR 0% Her2 3+  Ki67 60% | 6 x TCbHP  (docetaxe 75 mg/m², carboplatin AUC 6, trastuzumab loading dose 8 mg/kg, maintainance 6 mg/kg, pertuzumab loading dose 840mg, maintainance dose 420mg) q3w | No:  C2-C6 80% due to severe diarrhea | No | Yes |
| 22 | 77 | cT4b cN3a G2 | ER 100%  PR 90% Her2 3+  Ki67 30% | 6 x TCbHP  (docetaxe 75 mg/m², carboplatin AUC 6, trastuzumab loading dose 8 mg/kg, maintainance 6 mg/kg, pertuzumab loading dose 840mg, maintainance dose 420mg) q3w | Yes | Yes | Yes |
| 23 | 40 | cT3 cN1 G3 | ER 0%  PR 0% Her2 0  Ki67 70% | 12 x nab-paclitaxel 125 mg/m^2^ + carboplatin AUC 1.5 q1w, followed by 4 x epirubicin 90 mg/m^2^ + cyclophosphamid 600 mg/m^2^ dd q2w | Yes | Yes | Yes |
| 24 | 48 | cT1 cN1 G3 | ER 30%  PR 0% Her2 3+  Ki67 60% | 6 x TCbHP  (docetaxe 75 mg/m², carboplatin AUC 6, trastuzumab loading dose 8 mg/kg, maintainance 6 mg/kg, pertuzumab loading dose 840mg, maintainance dose 420mg) q3w | Yes | Yes | Yes |
| 25 | 57 | cT4d cN1 G3 | ER 0%  PR 0% Her2 0  Ki67 70% | 12 x nab-paclitaxel 125 mg/m^2^ + carboplatin AUC 1.5 q1w, followed by 4 x epirubicin 90 mg/m^2^ + cyclophosphamid 600 mg/m^2^ dd q2w + 3 x pembrolizumab 200mg q3w | Yes | Yes | Yes |
| 26 | 43 | cT2 cN3 G3 | ER 85%  PR 70% Her2 0 Ki67 80% | 12 x nab-paclitaxel 125 mg/m^2^ + carboplatin AUC 1,5 weekly, followed by 4 x epirubicin 90 mg/m^2^ + cyclophosphamid 600 mg/m^2^ dd q2w | Yes | No | No |
| 27 | 28 | cT2 cN1 G3 | ER 0%  PR 0% Her2 0 Ki67 60% | 12 x nab-paclitaxel 125 mg/m^2^ + carboplatin AUC 1.5 q1w, followed by 4 x epirubicin 90 mg/m^2^ + cyclophosphamid 600 mg/m^2^ dd q2w + 3 x pembrolizumab 200mg q3w | No:  discontinuation pembrolizumab after 3 doses (from C3 on) due to thyreoiditis, chemotherapy completed | No | Yes |
| 28 | 39 | cT1 cN1 G3 | ER 8%  PR 0% Her2 3+  Ki67 99% | 6 x TCbHP  (docetaxe 75 mg/m², carboplatin AUC 6, trastuzumab loading dose 8 mg/kg, maintainance 6 mg/kg, pertuzumab loading dose 840mg, maintainance dose 420mg) q3w | Yes | Yes | Yes |
| 29 | 68 | cT4 cN1 G3 | ER 60%  PR 0% Her2 3+ Ki67 60% | 6 x TCbHP  (docetaxe 75 mg/m², carboplatin AUC 6, trastuzumab loading dose 8 mg/kg, maintainance 6 mg/kg, pertuzumab loading dose 840mg, maintainance dose 420mg) q3w | Yes | Yes | Yes |
| 30 | 41 | cT1 cN1 G3 | ER 100%  PR 100% Her2 low (1+)  Ki67 70% | 4x epirubicin 90mg/m2 + cyclophosphamid 600mg/m2 dd q2w, followed by 12x paclitaxel 80mg/m2 q1w | Yes | No | Yes |
| 31 | 53 | cT1 cN1 G3 | ER 0%  PR 0% Her2 3+ Ki67 30-40% | 6 x TCbHP  (docetaxe 75 mg/m², carboplatin AUC 6, trastuzumab loading dose 8 mg/kg, maintainance 6 mg/kg, pertuzumab loading dose 840mg, maintainance dose 420mg) q3w | Yes | Yes | Yes |
| 32 | 36 | cT2 cN1 G2 | ER 100%  PR 80% Her2 low (2+, ISH neg.)  Ki67 25% | 4x epirubicin 90mg/m2 + cyclophosphamid 600mg/m2 dd q2w, followed by 12x paclitaxel 80mg/m2 q1w | Yes | No | Yes |
| 33 | 64 | cT2 (n=2) cN1 G3 | ER 60%;70%  PR 3%;0% Her2 3+ Ki67 30%; 20% | 6 x TCbHP  (docetaxe 75 mg/m², carboplatin AUC 6, trastuzumab loading dose 8 mg/kg, maintainance 6 mg/kg, pertuzumab loading dose 840mg, maintainance dose 420mg) q3w | Yes | Yes | Yes |
| 34 | 65 | cT2 cN1 G3 | ER 30%  PR 0% Her2 3+ Ki67 35% | 6 x TCbHP  (docetaxe 75 mg/m², carboplatin AUC 6, trastuzumab loading dose 8 mg/kg, maintainance 6 mg/kg, pertuzumab loading dose 840mg, maintainance dose 420mg) q3w | No:  only 3/6 cycles due to akute kidney injury grad 3 | Yes | Yes |
| 35 | 53 | cT2 cN1 G3 | ER 0%  PR 0% Her2 low (1+)  Ki67 80% | 12 x nab-paclitaxel 125 mg/m^2^ + carboplatin AUC 1.5 q1w, followed by 4 x epirubicin 90 mg/m^2^ + cyclophosphamid 600 mg/m^2^ dd q2w + 3 x pembrolizumab 200mg q3w | Yes | Yes | Yes |
| 36 | 67 | cT2 cN1 G3 | ER 0%  PR 0% Her2 3+ Ki67 90% | 6 x TCbHP  (docetaxe 75 mg/m², carboplatin AUC 6, trastuzumab loading dose 8 mg/kg, maintainance 6 mg/kg, pertuzumab loading dose 840mg, maintainance dose 420mg) q3w | No:  after C5 dosisreduction 80% due to diarrhea grade III with hypocalemia and acute kidney injury grade III | No | Yes |
| 37 | 49 | cT1 cN1 G3 | ER 85%  PR 1% Her2 3+ Ki67 35% | 6 x TCbHP  (docetaxe 75 mg/m², carboplatin AUC 6, trastuzumab loading dose 8 mg/kg, maintainance 6 mg/kg, pertuzumab loading dose 840mg, maintainance dose 420mg) q3w | Yes | Yes | Yes |
| 38 | 58 | cT1 cN1 G3 | ER 0%  PR 0% Her2 3+ Ki67 43% | 4x epirubicin 90mg/m2 + cyclophosphamid 600mg/m2 dd q2w, followed by 12x paclitaxel 80mg/m2 q1w | Yes | No | Yes |
| 39 | 52 | cT1 cN1 G3 | ER 0%  PR 0% Her2 low (2+, ISH neg.)  Ki67 50% | 4 x epirubicin 90 mg/m^2^ + cyclophosphamid 600 mg/m^2^ dd q2w, followed by 12 x nab-paclitaxel 125 mg/m^2^ + carboplatin AUC 1.5 q1w + 3 x pembrolizumab q3w | Yes | Yes | Yes |
| 40 | 46 | cT2 cN1 G3 | ER 0%  PR 0% Her2 3+ Ki67 30% | 6 x TCbHP  (docetaxe 75 mg/m², carboplatin AUC 6, trastuzumab loading dose 8 mg/kg, maintainance 6 mg/kg, pertuzumab loading dose 840mg, maintainance dose 420mg) q3w | Yes | Yes | Yes |
